# Effectiveness of three machine learning models for prediction of daily streamflow and uncertainty assessment

**DOI:** 10.1016/j.wroa.2024.100297

**Published:** 2024-12-28

**Authors:** Luka Vinokić, Milan Dotlić, Veljko Prodanović, Slobodan Kolaković, Slobodan P. Simonovic, Milan Stojković

**Affiliations:** a Institute for Artificial Intelligence R&D of Serbia, Fruškogorska 1, Novi Sad 21000, Serbia; b School of Civil and Environmental Engineering, University of New South Wales (UNSW), Sydney 2052, NSW, Australia; c Faculty of Technical Sciences, University of Novi Sad, Trg Dositeja Obraovića 6, Novi Sad 21000, Serbia; d Department of Civil and Environmental Engineering, The University of Western Ontario, London N6A5B9 ON, Canada

**Keywords:** TKAN, Uncertainty, Machine learning, Streamflow, Time series forecasting

## Abstract

This study evaluates three Machine Learning (ML) models—Temporal Kolmogorov-Arnold Networks (TKAN), Long Short-Term Memory (LSTM), and Temporal Convolutional Networks (TCN)—focusing on their capabilities to improve prediction accuracy and efficiency in streamflow forecasting. We adopt a data-centric approach, utilizing large, validated datasets to train the models, and apply SHapley Additive exPlanations (SHAP) to enhance the interpretability and reliability of the ML models. The results show that TKAN outperforms LSTM but slightly lags behind TCN in streamflow forecasting. TKAN demonstrated strong alignment with observed statistical parameters, achieving a Mean Absolute Error (MAE) of 5.799 m³/s and a Nash-Sutcliffe Efficiency (NSE) of 0.958, compared to MAE and NSE values of 8.865 m³/s and 0.942 for LSTM, and 5.706 m³/s and 0.961 for TCN, respectively. Multi-step forecasting revealed TKAN's robust performance up to a three-day forecast horizon, with a slight decline in accuracy as the forecast period extended. Uncertainty analysis indicated reasonable variance levels, with a mean 3-day forecast uncertainty of 35.02% at a 95% confidence level for TKAN, compared to 39.95% for LSTM and 28.46% for TCN. For a 7-day forecast, TKAN showed a mean uncertainty of 40.97%, compared to 45.01% for LSTM and 36.22% for TCN. By enhancing model transparency and improving datasets, this study significantly advances the integration of machine learning into hydrological forecasting, offering robust methods for developing adaptive water management systems in response to changing climate conditions.

## Graphical abstract

**Figure:**
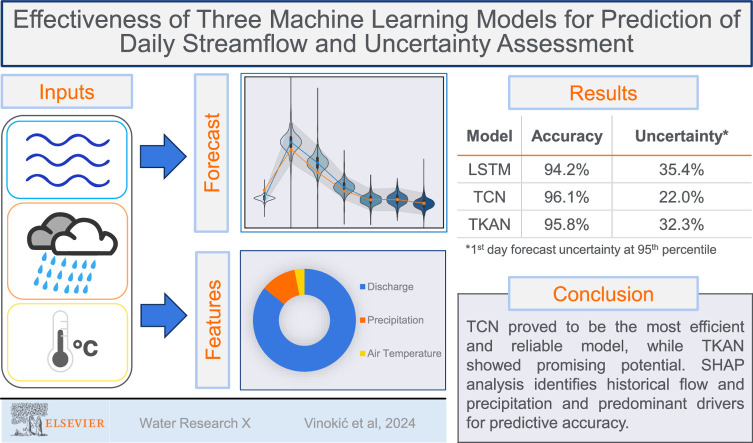

## Introduction

The complexity of hydrological systems, characterized by their dynamic interactions with climate factors, make streamflow prediction a major challenge (Mosavi et al., 2018). Traditional hydrological models, grounded in deterministic and stochastic approaches, have historically faced challenges in predicting streamflow, due to the intricate relationship between precipitation and runoff (Chang et al., 2002). This complexity is influenced by various factors, such as land use patterns, basin characteristics, soil moisture, vegetation, and human activities, among others, highlighting the limitations in models’ predictive capabilities (Hu et al., 2018). This emphasizes the necessity for innovative approaches to encapsulate the impact of the dynamic interaction of streamflow affecting variables (Zealand et al., 1999). The advancement in artificial intelligence (AI) and, more precisely, machine learning (ML) over the past few decades have made data-driven flow forecasting a viable option. Unlike traditional models, which are process-driven, ML models learn directly from data, enabling them to uncover complex, non-linear relationships that might not be immediately apparent (Zealand et al., 1999). Additionally, they require fewer input parameters, eliminating the challenges associated with complex parameters that can often be difficult to estimate in traditional process-driven models. This enables data-driven models to quickly deliver accurate results more efficiently, even with limited sets of variables.

Further development of computer processing power led to the improvement of Recurrent Neural Networks (RNNs), that are best suited for sequence modeling to capture dependencies and patterns in sequential data, such as time series, text, or speech, where the order of elements matter (Schmidt, 2019). For example, Long Short-Term Memory (LSTM), was specifically designed to store important information in long sequences without losing short-term predictive power (Hochreiter and Schmidhuber, 1997, Cojbasic et al., 2023, Dodig et al., 2024). The LSTMs have already shown remarkable results in capturing long-term temporal dependencies of hydrological time series data (Hu et al., 2018, Yuan et al., 2018, Arsenault et al., 2023). However, newer research suggests Temporal Convolutional Networks (TCNs) outperform LSTMs in sequence modeling (Bai et al., 2018, Lin et al., 2020). In recent years, more advanced ML approaches, such as transformer architectures, have also demonstrated significant potential to outperform LSTMs in time series forecasting (Hindersland), with generative pre-trained transformer successfully applied to predict fluid particle trajectories (Yang et al., 2023). However, one major limitation of transformers is their requirement for large dataset for effective training. This is particularly relevant to hydrological studies due to often limited data availability (often only daily data is recorded). Hence, transformers were found to have limited application in this study. More recently, one of the notable advances in Neural Network Architectures is the introduction of Kolmogorov-Arnold Networks (KAN) (Liu et al., 2024). This brand-new Neural Network architecture has generated a significant impact within the AI community, with Temporal KAN (TKAN) being used for time series forecasting in most recent studies (Vaca-Rubio et al., 2024, Xu et al., 2024). The latest research has shown that TKAN has great potential in time series forecasting, comparing the results with the already standard LSTM network (Genet and Inzirillo, 2024). Furthermore, studies have suggested the TKAN works well in multi-step forecasting, as it shows exceptional long-term forecasting capabilities (e.g., 15 time-steps in (Genet and Inzirillo, 2024)). However, most of the studies involving TKAN architecture have not been tested and validated on real-life physical phenomena, such as stream flows, that have a lot of measurement and physical uncertainties (e.g., inflows from tributaries, groundwater, snow melt, etc.).

Despite the promising advancements brought by machine learning models, their application in hydrological forecasting is not without challenges. Among the primary concerns are issues related to data scarcity and quality, model complexity, and the interpretability of machine learning model outputs. In particular, the optimization of model parameters, such as window size, has been shown to significantly impact the accuracy and reliability of forecasts (Shen et al., 2020). Furthermore, since the ML models are mostly “black box” models, it is quite difficult to estimate the uncertainties of model performances and predictions. Robust methods for understanding and interpreting the complex decisions made by ML models have recently been introduced through Explainable AI (XAI) techniques (Lundberg and Lee, 2017). However, there are only a few studies that incorporate these techniques in understanding the ML models outputs for hydrological predictions. Additionally, although decision-making processes benefit from incorporating uncertainty estimates for future predictions, this practice is often overlooked (Kompa et al., 2021).

In hydrology, uncertainties are ever-present, driving scientists and engineers to continuously improve estimation and mitigation techniques. Over the years, significant advancements have been made in quantifying the uncertainties of neural networks, with the suggestion that model performance is the best indicator of the discrepancy between model outputs and real-world processes, where uncertainty is represented by a prediction interval (Solomatine and Shrestha, 2009). Despite these advancements, the integration of uncertainty estimation within ML models remains rare. When applied, it is often performed only on the training dataset. Using this practice, uncertainty is evaluated based on data that the model has already seen and learned from, which may not offer a robust assessment of uncertainty, as it does not account for how the model might perform on unseen or new data.

Given the limited understanding of the optimization, explainability, and uncertainty of using ML models for future streamflow prediction, this study aims to provide the following research innovations: (1) reveal the predictive capabilities of TKAN, LSTM, and TCN models in hydrological forecasting, providing critical insights into their relative performance and suitability; (2) understand key prediction drivers through sensitivity analysis, exposing the inner workings of ML models and their influence on streamflow predictions; and (3) enhance understanding of prediction uncertainty with a conservative uncertainty analysis on test data, offering a more realistic assessment of model generalization to unseen conditions. To the best of our knowledge, this is one of the rare studies in the field of hydrology to address challenges related to data availability and model interpretability in streamflow prediction using machine learning, as well as to explore and understand the uncertainties associated with future predictions.

## Results and discussion

### Impact of historic window size selection

**Fig. 1:**
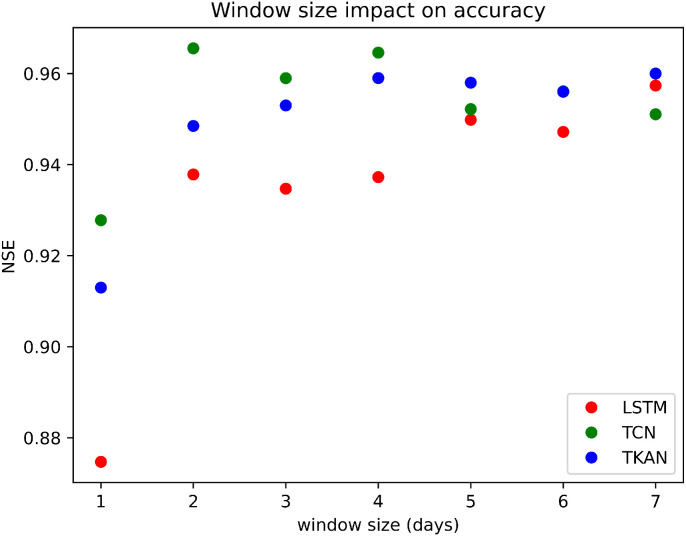
Comparison of window size impact on predictive accuracy for the test set.

Fig. 1 shows that the largest increase in accuracy happens when the historic data window size (how many previous events are used for prediction of future event) is increased from one to two days, where accuracy of LSTM increased by around 7% (from 0.875 to 0.94), and around 4% for TCN and TKAN (from 0.93 to 0.965 for TCN and from 0.91 to 0.95 for TKAN). This indicates that predicting future flows using streamflow from just one day before and weather data from the current time-step could be limited. LSTM shows an incline in accuracy as the window size is widened, while the TCN peaks in accuracy when the window size is selected to be around 2-4 days, after which it gradually declines (from 0.96 to 0.948). Perhaps this could be explained by the capability of the LSTM to capture long-term dependencies, while TCNs are known to successfully capture short-term dependencies (Ehteram and Ghanbari-Adivi, 2023). On the other hand, TKAN appears to find stability around 3-4 days, after which the NSE shows no significant changes (around 0.96).

The results of this analysis offer valuable insights into the impact of window size, highlighting the importance of selecting an optimal window size. Achieving a good balance requires providing enough input data without overwhelming the model with excessive information, while keeping the window size relatively small (Qiao et al., 2023). The difference in optimal window size across models underscores the importance of model-specific tuning of input parameters. The choice of window size also depends on specific physical and hydrological characteristics, such as river length, catchment area, and time of concentration. In this case, theoretically, since the catchment area and time of concentration are small, the two-day threshold offers more than enough data for high accuracy, which is in alignment with the results (Buttle, 1998). Additionally, similar window sizes have been utilized in prior research for daily streamflow forecasting (Rahimzad et al., 2021).

### Streamflow forecast examination

**Fig. 2:**
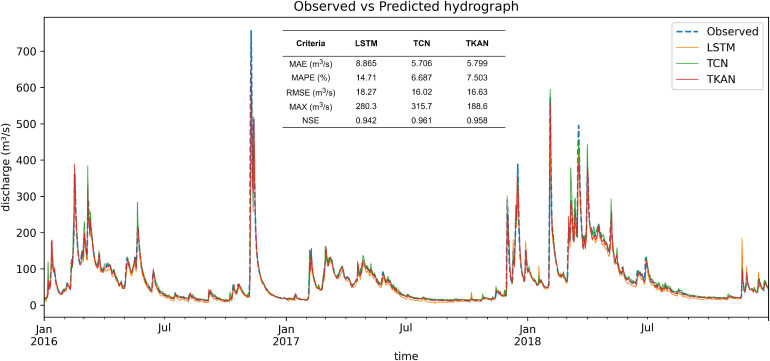
Time series of predicted streamflow compared to observed data for the test set.

Window size for the single-step (one day ahead) machine learning streamflow prediction models is chosen to be two, four, and five days for TCN, TKAN and LSTM respectively. For the test period, over the course of three years, from January 2016 to December 2018, the observed discharge rates show considerable fluctuation with several significant peaks corresponding to potential flood events. Fig. 2 indicates that all three models follow the observed trend with great accuracy, showing dynamic matching of historical streamflow time series with each of the model predictions.

There are instances, notably around the peak discharges, where all models slightly deviate from the observed data, indicating challenges in predicting extreme events. This suggests that data-driven models struggle predicting extremely rare events that are not represented in the training set. The maximum daily flow value in the test set occurred on the 9th of November 2016, measured at 756.3 m³/s. In comparison, the predictions from the machine learning models were 614.3 m³/s, 651.2 m³/s, and 679.7 m³/s for LSTM, TCN, and TKAN, respectively. However, the largest discrepancy occurred on the previous day, November 8th, 2016. The measured streamflow was 564.8 m³/s, while the predicted values were considerably lower: 292.3 m³/s for the LSTM, 202.0 m³/s for the TCN, and 289.4 m³/s for the TKAN. This event and possible origins of error are investigated thoroughly in later sections. Tabulated metrics show that TCN and TKAN produce smaller residuals on average, compared to the LSTM. TCN achieved the lowest error rates across most metrics, suggesting its enhanced capability in capturing the temporal dynamics of the system, although TKAN's maximum error is far lower compared to the other two models. This could be due to the adaptive activation function that adapts well to the extremes (Vaca-Rubio et al., 2024, Genet and Inzirillo, 2024). The accuracy of streamflow predictions using LSTM and TCN can be compared with previous studies, while the results for TKAN are novel in this field, marking its first application in streamflow forecasting. The LSTM model demonstrates similar accuracy, in terms of Nash-Sutcliffe Efficiency (NSE = 0.942), compared to the previous studies (e.g. NSE = 0.939 in (Rahimzad et al., 2021)), while TCN produces comparable results (NSE=0.961) with studies that utilized more granular data (e.g. NSE around 0.95 in (Lin et al., 2020)). However, direct comparisons are somewhat limited due to differences in study areas and data sources. Nonetheless, the accuracies of all three models suggest a significant improvement over simpler ANNs in similar conditions, where models achieved an NSE of less than 0.8 for monthly flow-rate forecast (Kostić et al., 2016).

**Fig. 3:**
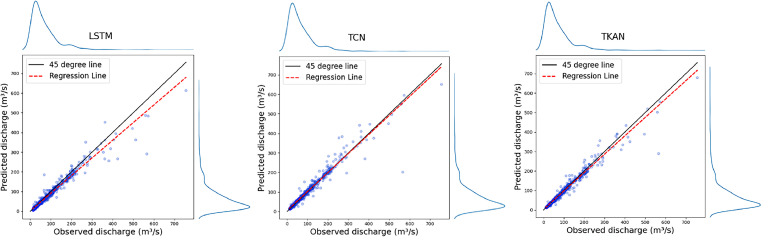
Scatter plots for each of the models, with regression line and a 45-degree line indicating a perfect correlation.

Fig. 3 shows scatter plots with a regression line for each of the models, comparing the predictions to observed values. All three plots exhibit a strong linear relationship between observed and predicted values, evidenced by the dense clustering of points along the 45-degree line. This clustering suggests that the models used for prediction are performing well, particularly for the central range of values. The presence of some points that fall well below the diagonal line indicates instances where the models' predictions significantly deviate from the observed data. These instances occurred in the prediction of the largest peak flow event of the test set, which will be explored in the following sub-section. Regression line indicates a slight underprediction, which may be attributed to an imbalance in the data, such as overrepresentation of lower values or insufficient coverage of higher values, leading to a model bias that skews predictions downward.

Scatter plots on Fig. 3 illustrate a better alignment with the observed data for the TKAN and TCN compared to the LSTM, demonstrating their superior performance in time series prediction, as evidenced in the previous research (Bai et al., 2018, Lin et al., 2020). Fig. 3 also shows that the regression line for TKAN and TCN follows the 45-degree line more closely in comparison to LSTM, indicating more evenly distributed residuals, with TCN showing near-perfect correlation, which is in accordance with the tabulated metrics.

#### Peak error exploration

Forecasted streamflow was evaluated against observed data, focusing on maximum daily flow (MDF - local maxima of observed daily-averaged flow) timing and magnitude. The largest discrepancy happened in the largest flood event of the test set, which occurred from Nov 7^th^ to around Nov 19^th^, 2016. This flood event is systematically examined to identify potential sources of errors (see Supplementary materials, section E, for hydrograph plots). MDF magnitude error varies from 5% to 15%, while MDF timing appears to be a miss for LSTM and match the observed for the TCN and TKAN. Even though all the models captured the timing of the MDF, and solidly predicted its magnitude, it appears that they couldn't quite capture a rapid change in streamflow that happened the day before the MDF. Further analysis of the rate of change for streamflow reveals that the mentioned instance is the 3^rd^ highest streamflow daily increase, with 755% increase from 75 to 565 m^3^/s. This means that the extreme changes (less than the 0.001 percentile) were underrepresented in the training set, indicating a potential area for model improvement with greater dataset. On the other hand, the problem might arise from poorly established gauge network, which leads to unaccounted-for excessive precipitation, which generates extreme streamflow, making it very hard for machine-learning models to predict it.

### Multi-step streamflow forecast capability and uncertainty analysis

To prioritize total volume accuracy over individual daily errors, multi-step forecasts are essential for water system management and decision-making. In this work, these forecasts are trained and tested solely on historical data, hence no uncertainty in precipitation data is considered.

The models generally perform well across the whole horizon, with great accuracy in the beginning of the horizon (up to the 3-days ahead), after which the NSE drops below 0.9 (see Supplementary materials, section C, for performance plot). However, these are also considered to be solid predictions ranging from 0.859 to 0.903. NSE of 0.915-0.946 suggests that TKAN outperforms LSTM, with a NSE of 0.894-0.928, while going toe-to-toe with TCN, with the NSE of 0.915-0.947 for the forecasts up to 3 days, but noticeably declines for the forecasts of streamflow for 4 days ahead to 0.888, highlighting challenges in capturing the nonlinear dynamics of hydrological processes over extended periods. While sharper decline of NSE for forecasts of 4 days ahead from 0.915 to 0.867 and 0.859 for TCN and TKAN, respectively, LSTM remains stable even after a 4-day horizon, falling from 0.894 to 0.875, showing the capability to adequately capture long-term dependencies. This, however, could simply be a product of randomness, since the relative difference between accuracy is around 2%.

The bootstrapping technique was applied to a sample of 100 randomly selected clusters, where the prediction for each day within a cluster was evaluated using the absolute percentage error (APE). The results show an expected increase in uncertainty as the forecast horizon extends (see Supplementary materials, section D, for uncertainty metrics). LSTM generally exhibited the highest uncertainty (median uncertainty of the first day prediction at 14% and 35% at the 95^th^ percentile), while TCN produced significantly better results, with median uncertainty of the first day prediction at 9% and 22% at 95^th^ percentile. To apply the uncertainty in practice, a prediction interval was calculated for a specific forecast.

**Fig. 4:**
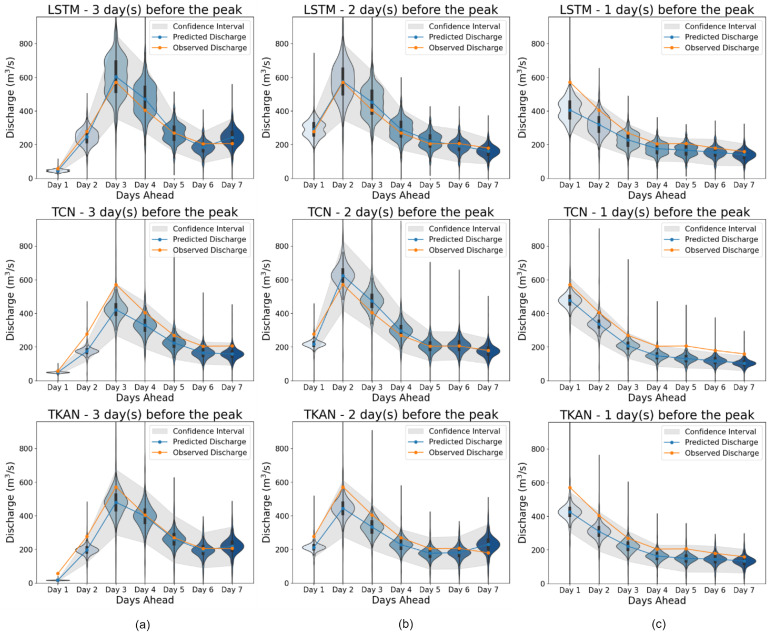
Multi-step forecast at three different time-steps for LSTM, TCN and TKAN with error probability plots for three different time-steps: (a) three days before the MDF, (b) two days before the MDF, and (c) one day before the MDF.

To assess the model's capability in anticipating MDFs, multi-step forecasts are examined. Fig. 4 shows one example of a MDF prediction, where the multi-step forecast can be seen with three different time-steps: (a) three days before the MDF, (b) two days before the MDF, and (c) one day before the MDF. As the MDF approaches, the uncertainty diminishes, allowing for increasingly accurate estimates with each passing day. Along with the uncertainty, an approximate illustration of potential error distribution is shown through the violin plots centered around the predicted values.

These results confirm that the MDF magnitude and timing are adequately captured by all three models, even with no knowledge of the flow of the actual previous days. Arguably, even more important metric is the total volume error. Total volumes are given through the hydrograph and compared with the recorded for the given flood occurrence. Volume errors (VE) are calculated based on the difference in water quantity that passes through the control section, the volumetric errors are calculated and are shown in Table 1.

**Table 1:** Total mean estimated volume of water in the 7-day horizon and volumetric error.

**Metrics**	**LSTM**	**TCN**	**TKAN**
Upper limit predicted volume	2745.9 m^3^	2334.2 m^3^	2432.8 m^3^
Total predicted volume	1947.3 m^3^	1749.4 m^3^	1708.0 m^3^
Lower limit predicted volume	1148.7 m^3^	1164.5 m^3^	983.1 m^3^
Total observed volume	2033.7 m^3^	2033.7 m^3^	2033.7 m^3^
Relative VE	9.13%	17.1%	24.2%
Max 95% confidence relative VE	48.3%	43.0%	51.7%

Assuming the implementation of these models is reservoir management with large enough capacity, volumetric error of a flood episode is of great importance. The largest relative volumetric discrepancy in this flood event varies from 9 to 24% for the three said models, which would allow the implementation of dynamic reservoir system management, considering the MDFs can be predicted ahead of time (Ahmad and Simonovic, 2000). Note that this time window was chosen because of the large MDF, which could lead to flooding. From the flood mitigation standpoint, maximum possible VE is calculated for the flood event estimation to indicate the worst-case scenario for underestimating a single flood event. It should also be noted that after a first day miss, the model can recalibrate and provide a more accurate prediction.

### Feature importance

**Fig. 5:**
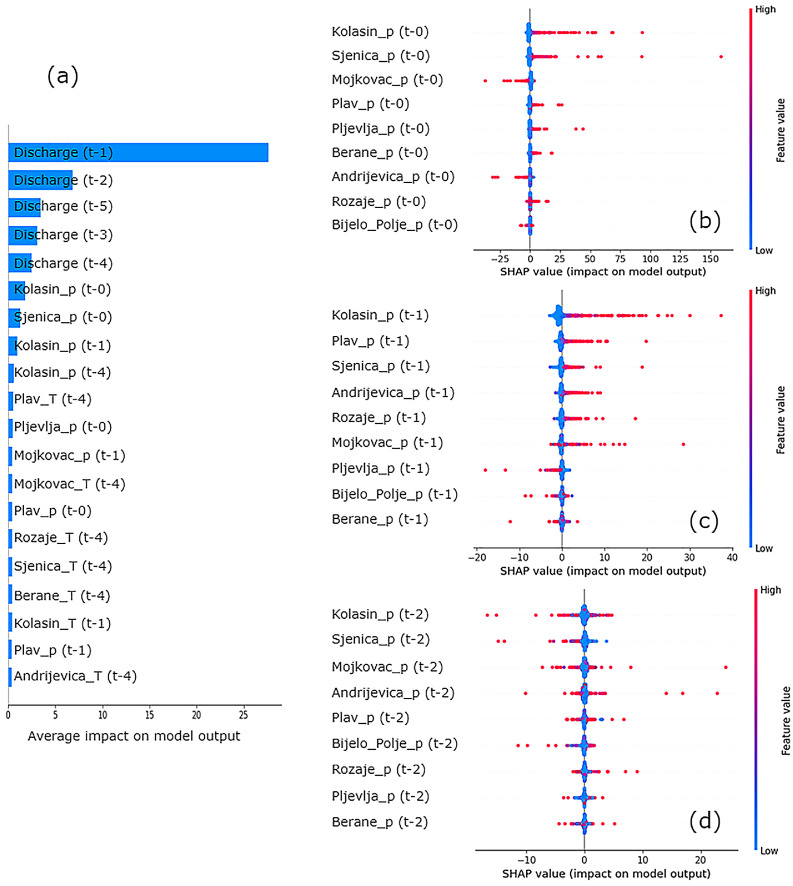
Feature importance shown trough (a) average feature impact on model outputs, and SHAP summary plots for precipitation impact from (b) today, (c) yesterday, and (d) the day before yesterday. The time-step notation for features is as follows: t-0 represents values for the prediction day (today), t-1 for yesterday, up to t-5 for five days prior. Historical streamflow data is given as discharge levels, while meteorological features, such as precipitation (p) and air temperature (T), include additional labels identifying their source (e.g., meteorological stations or precipitation gauges like Kolašin, Sjenica, and Plav).

SHAP analysis is used to determine the importance of different features in the forecasting models. For a broader understanding of the importance of features, the analysis was conducted within a window of 5 days (hydrological input of previous 5 days and meteorological input of previous 4 days and the day of the prediction). This way some of the longer dependencies can be attained, along with their significance in the models’ outputs. The following results are for the TCN model, as it demonstrated the best performance overall, making it the focus of the analysis. Feature importance plots are shown in Fig. 5. SHAP values indicate that recent discharge volumes are the most influential factor. However, precipitation also plays a critical role, considering the latter is the main driver in flood creation as a result of rainfall-runoff process (Kratzert et al., 2018). To investigate more closely into the importance of precipitation collected from each meteorological station. Figs. 5 (b) to (d) isolate the impact of precipitation from several previous time steps to explain the spatial effects of this variable.

Recent precipitation (*t-0* and *t-1*) has been shown to have the most impact on the model output aside from historic streamflow (discharge from *t-1* to *t-5*). In contrast, air temperature holds less significance, which aligns with expectations since it is a secondary exogenous variable. Nonetheless, air temperature contributes to enhanced prediction accuracy by influencing hydrological conditions, affecting processes such as snowmelt and evaporation rates. Due to these processes, there is a delayed hydrological response to changes in air temperature, justifying its inclusion on the feature importance list, particularly for data from four days prior. A wide spread in the SHAP values in Fig. 5 (d) suggests that the precipitation features have varying impacts randomly distributed on the model's predictions across different instances. The changes that these features bring should be considered as mainly noise, which is in alignment with the window size impact analysis, reaffirming that anything more than two time-steps (days) doesn't significantly contribute to the goodness of the model.

Considerable contributors, in terms of precipitation, are meteorological stations Kolašin, Sjenica, Plav and Andrijevica, with Kolašin appearing twice, impacting with precipitation from yesterday, as well as today, even though the gauge lays beside the basin, not in it. One explanation for this phenomenon is that the models extract information from this meteorological station to compensate for the areas where extreme precipitation is occurring, but not measured. This could indicate that large-scale rainfall was captured only in Kolašin (northern Montenegro), bypassing all the other measuring devices.

## Conclusion

This study marks a step forward in the application of machine learning for streamflow prediction, addressing vital aspects of model optimization, explainability, and uncertainty assessment. The comparative analysis of TKAN, LSTM, and TCN provides insights into their performance, establishing a comprehensive blueprint for model selection based on specific environmental and hydrological conditions. This research also contributes to improvements in understanding of model behavior through explainable AI and underscoring the importance of uncertainty quantification. Despite challenges posed by poorly established gauge networks which affect prediction accuracy during extreme conditions, the models demonstrate high levels of accuracy in both single-step and multi-step forecasts.

TKAN outperforms LSTM in both single-step and multi-step forecasts, while closely following TCN, which produced the highest accuracy and lowest uncertainty. Out of these three models, TCN proved the most successful and reliable neural network architecture for streamflow forecasting. Nevertheless, TKANs offer a novel perspective on AI problem-solving, with significant potential for further improvements. SHAP analysis identifies historical flow conditions and precipitation as predominant drivers for predictive accuracy, with temperature data indicating potential impacts from delayed processes (e.g. snowmelt).

This research highlights the need for ongoing enhancement of ML models in hydrology, with future studies set to delve deeper into refining these models for better adaptability and resilience in water resource management. Future studies should focus on enhancing the models' sensitivity to extreme events and integrating more comprehensive climatic variables to improve forecast reliability, as well as exploring more granular data integration and advanced model calibration techniques. Building on this, future research should aim to systematically evaluate and address uncertainty introduced by weather forecasts. Emphasis should be placed on development of robust frameworks capable of quantifying variability which would enhance the reliability of predictive models in operational scenarios.

## Materials and methods

**Fig. 6:**
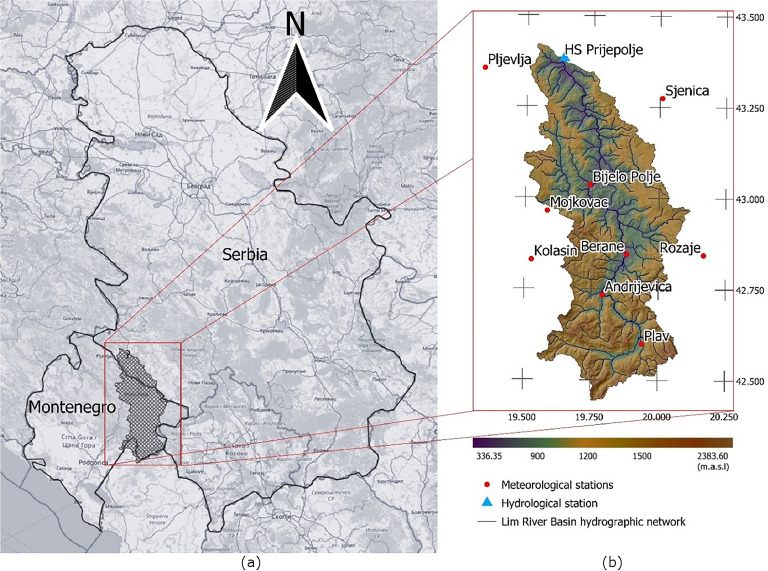
General methodology: streamflow forecasting.

The general methodology of this manuscript is structured around three primary phases: (1) dataset preparation; (2) developing and applying machine learning (ML) models and (3) prediction uncertainty estimation. In the first phase, ECMWF ReAnalysis v5 (ERA 5) dataset from European Centre for Medium-range Weather Forecast (ECMWF) is debiased to align with historical data, after which the two datasets are combined into a single multivariate time series. This complete dataset is then divided into subsets for training, validation, and testing the ML models for streamflow prediction based on several previous time-steps defined by window size. After the training stage is completed, models go through a performance evaluation based on common criteria (MAE, MAPE, RMSE, NSE), with uncertainty analysis performed on each of the already trained models for the test set. The general methodology can be seen in Fig. 6.

Daily forecasted streamflow is examined for the test period, which includes the standard statistical analysis in the terms of distribution comparison with correlation function through a scatter plot, along with evaluation criteria for each of the models. This helps identify the largest error, after which the cause of the error is investigated. After this, multi-step forecast capability of the models is evaluated. Daily peak timing, magnitude, and volume errors are calculated for the highest Maximum Daily Flow (MDF) in the test set of multi-step forecasting. Finally, input effects are also tested, with inspecting the window size impact on results, as well as SHAP analysis (feature importance).

### Study area

**Fig. 7:**
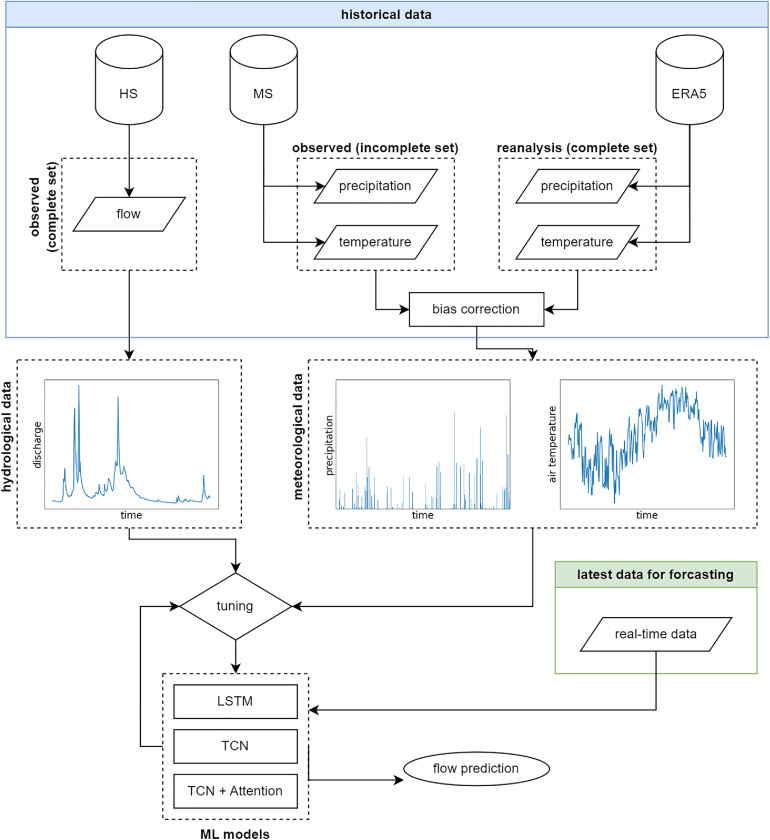
A map of the (a) regional and (b) local scale of the Lim River basin.

The case study was conducted on the part of the Lim River basin, positioned upstream of the Prijepolje hydrological station. The Lim River is the largest tributary of the Drina River, stretching over 201.6 kilomet**re**s (Stojkovic and Simonovic, 2020). It flows through three Southeastern European countries, starting in Montenegro, then Serbia, and finally Bosnia and Herzegovina, where it joins the Drina River. The Lim River basin involves the Lim water system, a multipurpose system, primarily used for hydropower generation, making the tributary significant (Stojkovic and Simonovic, 2019). The catchment area of the Lim River basin is approximately 3160 square kilometers and is mostly mountainous (Prohaska et al., 2004). The study area is shown in Fig. 7, with the regional scale on the left, and local scale on the right.

### Data collection and processing

#### Data availability

Gauged data comprises of hydrological and meteorological daily records that are available for different periods from 1949 to 2018. The Lim River flow rate is measured on automatic Prijepolje hydrological station (h.s.), while meteorological variables, precipitation, and air temperature, are measured on 9 meteorological stations (MS) on or in the proximity of the Lim River basin, selected based on Thiessen polygons, in order to get an appropriate spatial covering. Gauged data is gathered from the Republic HydroMeteorological Service of Serbia (RHMSS) and the Institute of HydroMeteorology and Seismology (IHMS) with the outline of the meteorological data shown in Table 2 (RHMSS, 2024, IHMS, 2024, Zdravković et al., 2014).

**Table 2:** Basic information for meteorological stations from which the data is gathered.

MS	latitude	longitude	Elevation (m.a.s.l.)	p_annual_ (mm)	T_annual_ (°C)	available data
Sjenica	43.27	20.00	1026	731.1	6.4	100%
Kolašin	42.82	19.52	1016	2019.2	7.5	100%
Pljevlja	43.36	19.36	761	787.1	8.7	100%
Bijelo Polje	43.03	19.75	720	898.2	9.4	45%
Plav	42.60	19.95	945	1218.0	6.9	21%
Rožaje	42.84	20.17	1014	935.8	/	21%
Mojkovac	42.96	19.58	894	1703.7	/	21%
Andrijevica	42.73	19.79	750	1089.0	/	21%
Berane	42.85	19.88	663	906.7	9.5	19%

Despite the Lim River basin being an important area, it is poorly gauged, and the stations are inadequately maintained, leading to insufficient observed data with gaps for certain periods. To resolve this issue, data from the ERA5 dataset is used to fill the gaps. Although additional meteorological data is needed to fill the gaps, complete hydrological time series is available for the whole considered period (1949-2018).

#### Global integrated weather systems

Reanalysis systems operate by merging the irregular observations and models that involve physical processes in order to create an integrated estimate of climate variable data (Sun et al., 2018). ERA5 is the fifth generation ECMWF reanalysis with available hourly data from 1940, and spatial resolution 0.25° x 0.25°, which is approximately 28km x 28km (Complete ERA5 global atmospheric reanalysis 2024). From this reanalysis, total precipitation and 2-meter air temperature are re-gridded onto the coordinates of the aforementioned meteorological stations. This is achieved with inverse distance weighting (IDW) between a station location and the four nearest centers of the reanalysis grid (shown as crosses in Fig. 7), after which a spatial interpolation is performed.

Eq. (1) is used to determine the weights of neighboring center points, while Eq. (2) calculates the interpolated value of a variable $\overset{⌣}{x}$ for a given x for 4 nearest neighbors:

(1) $$w_{j}=\frac{{d_{j}}^{-\alpha }}{\sum_{j}{d_{j}}^{-\alpha }};$$

(2) $$\overset{⌣}{x}=\;\sum_{j}w_{j}x_{j};$$

where $w_{j}$ represents weight of j-th center point, $d_{j}$ its distance, and α denotes an order of an inverse distance (Atiah et al., 2023). For this study, inverse squared distance is used, which means that α=2.

Initially, considering the availability of only directly measured data, and with the requirement for a minimum of 20 years of time series data for statistical robustness, only three meteorological stations with a continuous data record of 25 years meet the criteria for inclusion. However, by integrating gap-filled observations and reanalysis data, the dataset expands significantly, by approximately tenfold, making it substantially more suitable for the training set of ML models. It is crucial to adjust the reanalysis data to closely align with the observed data, minimizing biases. This adjustment process results in a "complete" dataset characterized by extensive coverage and consistent quality over a prolonged timeframe.

#### Bias correction

One of the crucial points of this methodology is to bridge the gap between the empirical specificity of observed data and comprehensive coverage of reanalysis data through the data-centric approach. A pragmatic technique, delta change, is utilized for bias correction of precipitation data from ERA 5 reanalysis. More accurately, a principle of equaling the mean monthly precipitation sums is used, as follows:

(3) $$\psi \left(month\right)=\frac{{\bar{x}}_{monthly}^{obs}}{{\bar{x}}_{monthly}^{ERA\;5}},$$

(4) $${\hat{x}}^{ERA\;5}=\psi *x^{ERA\;5}.$$

The Eq. (3) is used to calculate the transfer function ψ for the bias correction, where ${\bar{x}}_{monthly}^{obs}$ and ${\bar{x}}_{monthly}^{ERA\;5}$ represent the monthly mean of gauged and variable from the reanalysis dataset respectfully, while Eq. (4) shows the implementation of transfer function on ERA 5 time series data $x^{ERA\;5}$, generating a bias corrected series ${\hat{x}}^{ERA\;5}$. This simplistic approach is used to address systematic biases in the reanalysis data. By applying this correction factor, monthly averages, as well as total precipitation sums, are ensured to align with the observed.

### Machine-learning models

The following section provides a brief overview of the mathematical framework that forms the foundation of models, elaborating on the key concepts and principles that guide their development and application. The models used in this study include LSTM, TCN and TKAN. The training process of machine learning models requires careful consideration of the train-validation split strategy, as well as the choice of window size.

#### LSTM

The LSTM (Long Short-Term Memory) is a type of recurrent neural network (RNN) architecture designed to address the vanishing gradient problem encountered in traditional RNNs (Hochreiter and Schmidhuber, 1997). Its cell comprises of a memory cell, input gate, forget gate, and output gate, collectively facilitating information retention and updating over sequential data. The memory cell selectively preserves long-term dependencies from prior time steps, while the input gate governs new information influx. Conversely, the forget gate manages the retention of past information, and the output gate determines the data forwarded to subsequent time steps or utilized for prediction. The LSTM excels at capturing temporal dependencies and memory effects in time series e.g. hydrological processes, such as the delayed response of runoff to precipitation events (Kratzert et al., 2018).

#### TCN

Temporal Convolutional Networks (TCNs) are a type of neural network architecture designed for sequence modeling tasks (Lea et al., 2016). TCNs leverage 1D convolutional layers with dilated convolutions to efficiently capture temporal dependencies within input sequences. By using causal convolutions, TCNs ensure that predictions only depend on past or present information, maintaining causality in the model. Additionally, TCNs incorporate residual connections to facilitate the training of deeper networks and thus mitigate the vanishing gradient problem. With their ability to capture both short-term and long-term dependencies, TCNs have proven effective in time series forecasting tasks.

#### TKAN

Kolmogorov-Arnold Networks (KANs) are a type of neural network inspired by the Kolmogorov-Arnold representation theorem, differing from traditional Multi-Layer Perceptions (MLPs) by having learnable activation functions on the edges instead of fixed activations at nodes (Liu et al., 2024). In KANs, each connection between nodes is a learnable univariate function, often a spline, eliminating traditional linear weight matrices, which allows KANs to flexibly represent complex mappings with fewer parameters and simplified node operations, only containing summations (Liu et al., 2024). Temporal Kolmogorov-Arnold Networks represent modified KANs better suited for time series analysis, integrating aspects of RNNs by introducing the time dependency to transformation function to manage temporal dependencies effectively (Genet and Inzirillo, 2024).

#### Training-validation-test split

**Fig. 8:**
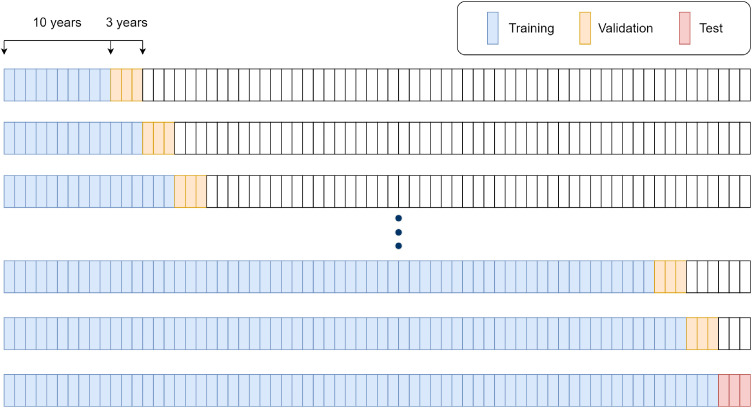
Expanding window operation scheme.

The training and validation of the models are done in accordance with the expanding window operations. Firstly, the models are trained for the 10-year block and validated based on the following 3-year block of the dataset. After this, the previous validation set becomes part of the training set, while the validation moves to the next 3-year block, expanding the window of time series data based on which the models trained on. The final model is then trained with data from the whole dataset excluding the test set, which is the last 3-year block, from 2016 to 2018. Fig. 8 illustrates how expanding window operation works, with 1 block representing 1 year worth of data.

#### Input window size

**Fig. 9:**
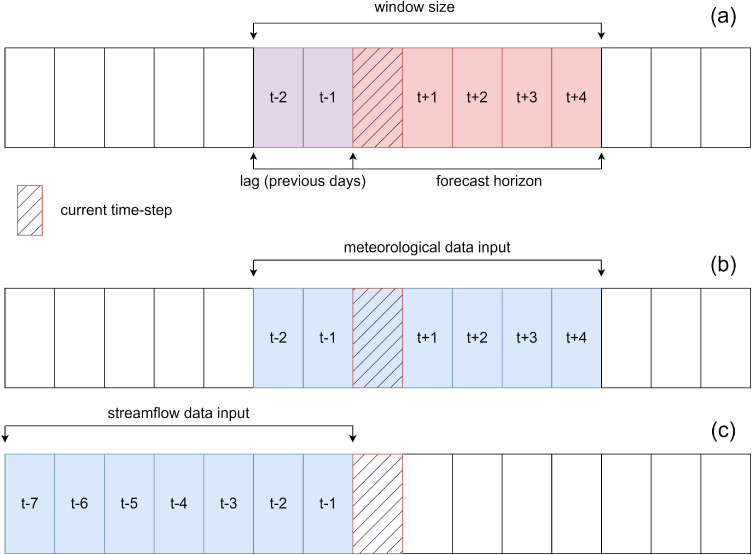
Example of (a) input window size formation, (b) meteorological data input, and (c) streamflow data input for a lag of 2 time-steps and forecast horizon of 5 time-steps.

Input window size represents a sum of lag (number of past time-steps used) and forecast horizon (number of future time-steps in the forecast). This is defined according to meteorological data requirements, since the input data needed for a prediction include weather forecast data for the forecast horizon. When the window size is defined, the same size is used to select input for previous streamflow data. For example, if the forecast horizon is 5 days and lag is set to be 2 days, the input data determined by window size is illustrated in Fig. 9. For single-step predictions, window size refers to the amount of streamflow past time-steps considered (*n*), which involves using hydrological data from the previous *n* time-steps and meteorological data from the last *n-1* time-steps, along with data from the current time-step. Collectively, these contribute to a total of *n* time-steps, defining the window size.

Window size is systematically varied to examine how it affects the accuracy of the model and its ability to make reliable predictions, providing a better understanding of how to optimize time series forecasting models. To observe the impact on accuracy, which is represented through NSE, various window sizes were inspected, from 1 day up to 7 days, beyond which the size would be unreasonably large.

### Model performance evaluation criteria

Model performance is evaluated via a wide range of criteria to ensure a comprehensive assessment of accuracy and efficiency. Mean Absolute Error (MAE) offers a straightforward measure of the average magnitude of errors between predicted and observed values, without considering their direction. The calculation is performed using the following formula:

(5) $$MAE=\frac{\sum_{i}^{n}\left|Q_{i}^{mod}-Q_{i}^{obs}\right|}{n},$$

where $Q_{i}^{mod}$ and $Q_{i}^{obs}$ represent the predicted and measured discharge for i-th time-step respectfully, while n denotes the number of time-steps. Mean absolute percentage error (MAPE), on the other hand, provides an insight of the mean magnitude of predicted error relative to observed values:

(6) $$MAPE=\frac{\sum_{i}^{n}\left|\frac{Q_{i}^{mod}-Q_{i}^{obs}}{Q_{i}^{obs}}\right|}{n}.$$

Root Mean Square Error (RMSE) provides a different perspective, emphasizing larger errors more significantly due to its squaring function, conveyed through this equation:

(7) $$RMSE=\sqrt{\frac{\sum_{i}^{n}{\left(Q_{i}^{mod}-Q_{i}^{obs}\right)}^{2}}{n}}.$$

Maximum error (MAX) captures the largest single discrepancy between a predicted and measured value, highlighting the worst-case scenario in prediction accuracy.

Lastly, the Nash-Sutcliffe Efficiency (NSE) metric is employed to assess the predictive accuracy of hydrological models by comparing the observed and predicted values, where a value of one indicates a perfect match between model predictions and observations, thereby offering a nuanced view of model performance in simulating specific phenomena. This metric is calculated through the following expression:

(8) $$NSE=\frac{\sum_{i}^{n}{\left(Q_{i}^{obs}-Q_{i}^{mod}\right)}^{2}}{\sum_{j}^{n}{\left(Q_{i}^{obs}-{\bar{Q}}^{obs}\right)}^{2}},$$

where ${\bar{Q}}^{obs}$ denotes the mean measured discharge for the period. Together, these criteria provide a multifaceted framework for evaluating the performance of models across various dimensions of accuracy and predictive quality.

Note that some of these performance evaluation criteria refer to certain kinds of error; this is retained as it is the original name of the said evaluation methods. However, instead of error, discrepancy or difference might be a more appropriate term, given that the accuracy of measured streamflow isn't absolute.

### Uncertainty analysis

The uncertainty analysis is frequently absent in ML modeling, especially in time series analysis, but it is something that should be investigated and regularly presented along with the results of the model output. In this study, a type of bootstrapping method is used to estimate uncertainty in multi-step streamflow forecast. The uncertainties are expressed using Prediction Intervals (PI) rather than Confidence Interval (CI), since they indicate a discrepancy between the outputs of the models and observed historical records for a specific prediction (Solomatine and Shrestha, 2009). This way, the uncertainties also refer to inherent randomness in real-world processes, leading to a more comperhensive representation of uncertainty in model-based prediction.

**Fig. 10:**
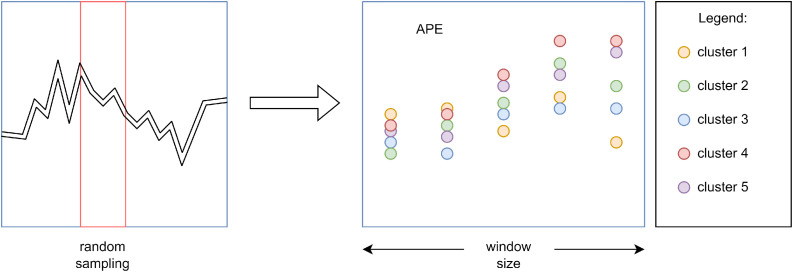
Illustration of bootstrapping technique applied in predictive uncertainty analysis.

Upon completion of the model training, a cluster of a certain window size is extracted from the test set through random sampling, after which an error estimation is performed and stored for the acquired prediction (Didona and Romano, 2014). Repeating this process n times results in n-cluster empirical distribution, where Kernel Density Estimation (KDE) is used to smooth the distributions for each prediction on the horizon. The models use a 7-day horizon for multi-step forecasting, making the result of bootstrapping a set of seven violin plots that visually represent the outcomes for each day. Finally, the PI is computed through simple statistical calculation based on confidence level (defining prediction range), described by the following formula:

(9) $$\begin{array}{c} \mathrm{PI}=x\pm t_{\alpha /2}\varepsilon , \end{array}$$

where x represents the value of a specific prediction, $t_{\alpha /2}$ denotes t-value from the t-distribution for a certain confidence level (95% confidence level is used), and *ε* represents the error term. Absolute Percentage Error (APE) is used for this because it intuitively expresses errors in percentage terms, making it a natural choice for evaluating forecast accuracy. Cluster extraction procedure from the test set is shown in Fig. 10.

This analysis estimates uncertainty, which can be divided into two categories: aleatoric and epistemic. Aleatoric uncertainty represents inherent noise that cannot be reduced, while epistemic uncertainty, stemming from a lack of knowledge, can be minimized with additional information about the models (Hüllermeier and Waegeman, 2021).

### Model parameter assessment (Explainable AI)

To further explore and explain the outputs of the machine learning models, Shapley Additive Explanations (SHAP analysis) is conducted. SHAP is a robust method for interpreting predictions from complex machine learning models by assigning an importance value to each feature. Based on cooperative game theory, particularly Shapley values, SHAP ensures fair attribution of each feature's contribution to a prediction (Lundberg and Lee, 2017). This method harmonizes several existing explanation approaches, ensuring properties like local accuracy, missingness, and consistency across various model types, making it an essential tool for enhancing model transparency and trustworthiness (Lundberg and Lee, 2017). This approach allows for detailed insights into how changes in individual features impact the outputs of the models (Manojlović et al., 2022).

## CRediT authorship contribution statement

**Luka Vinokić:** Writing – original draft, Visualization, Methodology, Investigation, Formal analysis, Data curation. **Milan Dotlić:** Writing – review & editing, Validation, Software, Methodology, Formal analysis. **Veljko Prodanović:** Writing – review & editing, Supervision, Methodology, Conceptualization. **Slobodan Kolaković:** Writing – review & editing. **Slobodan P. Simonovic:** Writing – review & editing. **Milan Stojković:** Writing – review & editing, Supervision, Methodology, Conceptualization.

## Declaration of competing interest

The authors declare that they have no known competing financial interests or personal relationships that could have appeared to influence the work reported in this paper.

## Data Availability

Data will be made available on request.

## Appendix Supplementary materials

Image, application 1
