# Supplementary material for: Effectiveness of three machine learning models for prediction of daily streamflow and uncertainty assessment
Source: Water Res X. 2024 Dec 28;27:100297. doi: 10.1016/j.wroa.2024.100297 (PMC11764612; doi:10.1016/j.wroa.2024.100297)
Supplement: Supplementary file 1 [file mmc1.docx]

SUPPLEMENTARY MATERIALS

A Inverse Distance weighting illustration


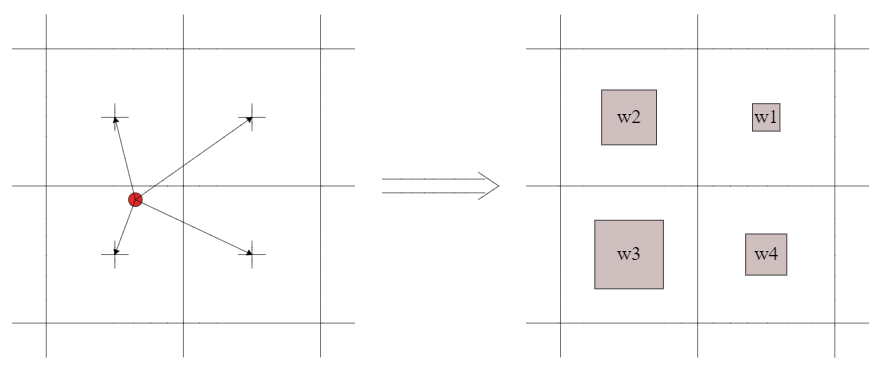


**Figure S 1.** Inverse distance weighting scheme.

B Boxplots for the test set


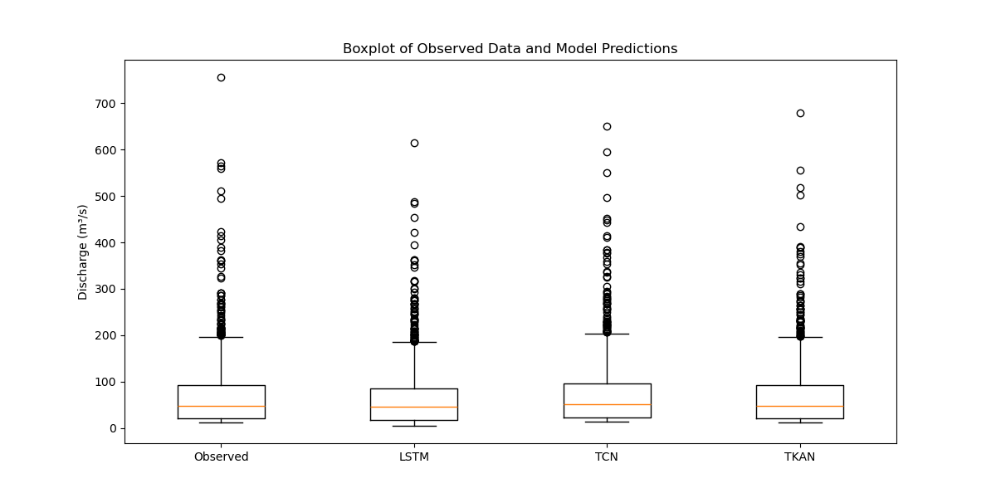


**Figure S 2.** Comparison of boxplots for observed data and outputs of the models.

C Multi-step forecasting capability


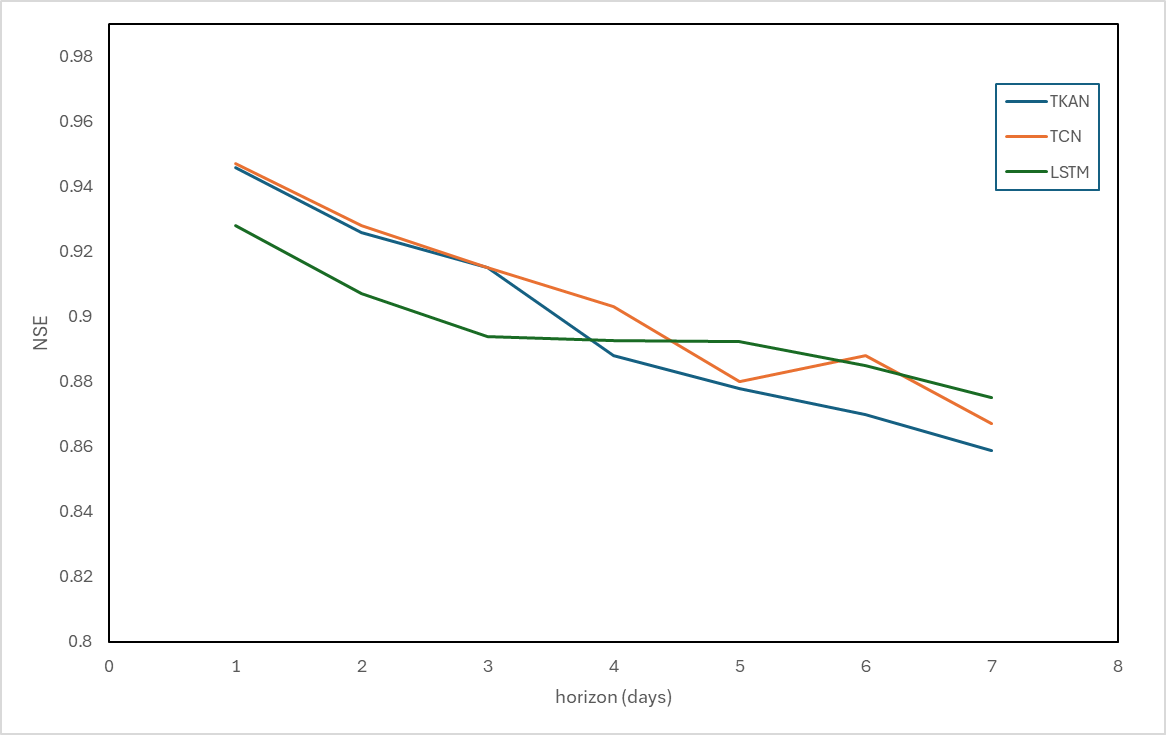


**Figure S 3.** Model accuracy for different forecast horizons.

D Uncertainty for a 95% confidence level

**Table S 1.** Median uncertainty and uncertainty for a 95% confidence level (in percentage).

| **Day of the horizon** | **LSTM** | | **TCN** | | **TKAN** | |
| --- | --- | --- | --- | --- | --- | --- |
|  | **50%** | **95%** | **50%** | **95%** | **50%** | **95%** |
| 1^st^ | 14.31% | 35.38% | 9.52% | 21.96% | 8.99% | 32.27% |
| 2^nd^ | 16.45% | 39.39% | 10.69% | 29.37% | 11.65% | 33.00% |
| 3^rd^ | 17.59% | 45.07% | 12.63% | 34.04% | 14.09% | 39.79% |
| 4^th^ | 17.54% | 45.04% | 13.52% | 41.00% | 14.74% | 44.27% |
| 5^th^ | 17.64% | 47.03% | 14.50% | 42.53% | 15.30% | 40.82% |
| 6^th^ | 17.10% | 51.00% | 14.92% | 40.04% | 16.72% | 48.68% |
| 7^th^ | 18.00% | 52.12% | 15.69% | 44.57% | 18.13% | 47.98% |

E Largest peak estimation


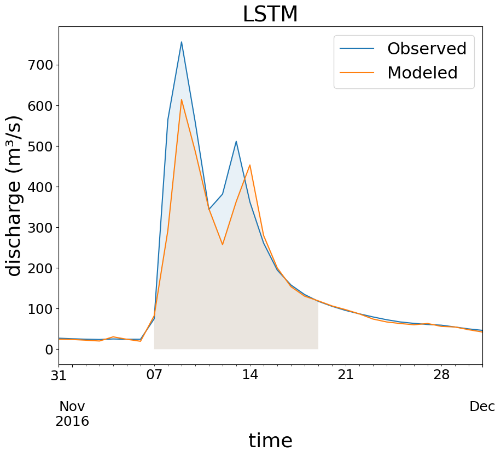

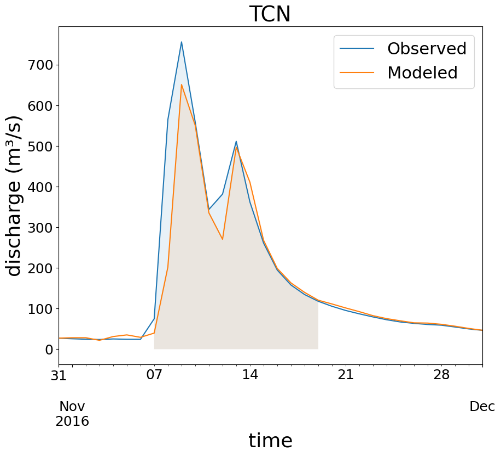

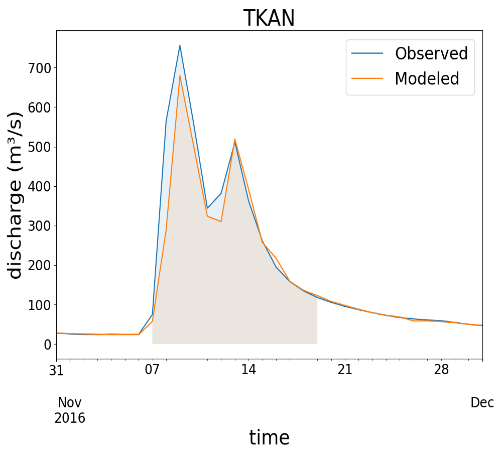


**Figure S 4.** Largest peak hydrograph volume compared to the corresponding hydrograph in the outputs of the models.
